# Prevalence of Hypertension in China: A Cross-Sectional Study

**DOI:** 10.1371/journal.pone.0065938

**Published:** 2013-06-11

**Authors:** Yun Gao, Gang Chen, Haoming Tian, Lixiang Lin, Juming Lu, Jianping Weng, Weiping Jia, Linong Ji, Jianzhong Xiao, Zhiguang Zhou, Xingwu Ran, Yan Ren, Tao Chen, Wenying Yang, for the China National Diabetes and Metabolic Disorders Study Group

**Affiliations:** 1 Department of Endocrinology, West China Hospital, Sichuan University, Chengdu, People’s Republic of China; 2 Department of Endocrinology, Fujian Provincial Hospital, Fuzhou, People’s Republic of China; 3 Department of Endocrinology, Chinese People’s Liberation Army General Hospital, Beijing, People’s Republic of China; 4 Department of Endocrinology, Sun Yat-sen University Third Hospital, Guangzhou, People’s Republic of China; 5 Department of Endocrinology, Shanghai Jiaotong University Affiliated Sixth People’s Hospital, Shanghai, People’s Republic of China; 6 Department of Endocrinology, Peking University People’s Hospital, Beijing, People’s Republic of China; 7 Department of Endocrinology, China–Japan Friendship Hospital, Beijing, People’s Republic of China; 8 Department of Endocrinology, Xiangya Second Hospital, Changsha, People’s Republic of China; University of Hong Kong, China

## Abstract

**Aims:**

The present study aimed to assess the prevalence of hypertension among Chinese adults.

**Methods:**

Data were obtained from sphygmomanometer measurements and a questionnaire administered to 46239 Chinese adults ≥20 years of age who participated in the 2007–2008 China National Diabetes and Metabolic Disorders Study. Hypertension was defined as blood pressure ≥140/90 mm Hg or use of antihypertensive medication.

**Results:**

A total of 26.6% of Chinese adults had hypertension, and a significantly greater number of men were hypertensive than women (29.2% vs 24.1%, p<0.001). The age-specific prevalence of hypertension was 13.0%, 36.7%, and 56.5% among persons aged 20 to 44 years (young people), 45 to 64 years (middle-aged people), and ≥65 years (elderly people), respectively. In economically developed regions, the prevalence of hypertension was significantly higher among rural residents than among urban residents (31.3% vs 29.2%, p = 0.001). Among women or individuals who lived in the northern region, the disparity in the prevalence of hypertension between urban and rural areas disappeared (women: 24.0% vs. 24.0%, p = 0.942; northern region: 31.6% vs. 31.2%, p = 0.505). Among hypertensive patients, 45.0% were aware of their condition, 36.2% were treated, and 11.1% were adequately controlled.

**Conclusions:**

The prevalence of hypertension in China is increasing. The trend of an increase in prevalence is striking in young people and rural populations. Hypertension awareness, treatment, and control are poor. Public health efforts for further improving awareness and enhancing effective control are urgently needed in China, especially in emerging populations.

## Introduction

Cardiovascular and cerebrovascular diseases are the leading cause of death in China [1]. Most cardiovascular and cerebrovascular diseases are closely associated with hypertension [1]–[3]. Hypertension has become the most common and preventable risk factor for cardiovascular and cerebrovascular diseases worldwide [1], [4].

With economic growth and the urbanization of China, the prevalence of hypertension has changed in the past several decades, especially during the last 30 years. For example, a national-wide survey of hypertension in 1991, completed by a total sample population of 950356, aged 15 years and older, showed that the age-adjusted prevalence and absolute number of people with hypertension were 11.26% and approximately 94 million, respectively [5]. The estimated number of people with hypertension had increased by almost 60% compared with 1980 results [6]. After approximately 10 years, the China National Nutrition and Health Survey (NNHS) 2002, involving a nationally representative sample of approximately 141 892 adults, reported that 18%, or approximately 153 million Chinese adults aged 18 years and older, had hypertension [7]. This survey also showed that in China, between 1992–2002 more than 260 million people became overweight or obese, which increased the risk for hypertension, type 2 diabetes, dyslipidemia, and coronary heart disease [8]. Therefore, we hypothesize that with the growing modernization of China, the prevalence of hypertension will continue to increase.

Since the 1980 s, the economic transition in China has provoked sweeping changes in lifestyle involving overconsumption of dietary fat and reduction in physical activity, which have undoubtedly contributed to the increase in body weight [8], [9]. Such lifestyle changes are affecting young people in China. The proportion of children aged 7 to 18 years who were obese and overweight increased 28-fold between 1985–2000 [10]. Therefore, it is likely that the prevalence of hypertension may be dramatically rising among young people. Additionally, compared with developed regions, developing counterparts have more remarkable changes in lifestyle along with economic development [11]. In the last two decades, the rate of obesity has tripled in developing countries [12]. In developing nations, obesity is often most prevalent in wealthier sections of the population [13]. Considering the disparities in regional development, and the rapid development of the rural economy over the recent years in China, the problem of obesity and obesity-related illnesses, including hypertension, may be worsening at a dramatic rate in developing or rural areas. Previous studies have documented that there is a higher prevalence of hypertension in northern China compared with southern China, largely because of a greater body mass index (BMI), higher dietary salt intake, and other lifestyle factors among residents in the north [14]. However, it is unknown whether geographic variations in the prevalence in hypertension have changed over recent years because of lifestyle changes in Chinese adults.

To provide current and reliable data on the prevalence and epidemiological characteristics of selected cardiovascular disease risk factors, including hypertension, the China National Diabetes and Metabolic Disorders Study was conducted from June 2007 through to May 2008 in Chinese adults aged 20 years and older [15].

## Methods

### Study Participants

We studied the participants who had been recruited to the China National Diabetes and Metabolic Disorders Study, of which full details are described by Yang et.al [15]. In brief, a multistage stratified sampling method was used to select a nationally representative sample of the general population aged 20 years or older in China. A total of 54,240 people were selected and invited to participate in the survey. A total of 47,325 people (18,976 men and 28,349 women) completed the study. The overall response rate was 87.3%(81.0% for men and 92.0% for women; 88.1% in urban areas and 82.7% rural areas). After the exclusion of 538 persons for whom demographic information was missing and 548 for whom data on fasting or 2-hour plasma glucose levels were missing, 46,239 adults were included in the final analysis.

The Medical Research Ethics Committee of the China–Japan Friendship Hospital reviewed and approved the present study. The written informed consent was obtained from each participant before data collection.

### Data Collection

Data collection was conducted in examination centers at local health stations or in community clinics in the participants’ residential area. Details of data collection, including biometric and biochemical characteristics, were presented in our previous paper [15]. Additionally, blood samples of participants were taken at 0, 30, and 120 minutes in an oral glucose-tolerance test (OGTT) and then the serum was separated and stored frozen at −80°Cfor insulin measurements. All the serum insulin was measured by radioimmunoassay in the laboratory of China–Japan Friendship Hospital. To investigate the relationships between blood pressure and insulin resistance and/or hyperinsulinemia, the homeostasis model assessment of insulin resistance (HOMA-IR) [16] was calculated and used to estimate insulin sensitivity, and insulin area under-curve (IAUC) [17] was calculated and employed to estimate hyperinsulinemia.

1999 World Health Organization diagnostic criteria was used to diagnose diabetes, impaired fasting glucose and impaired glucose tolerance [18]. Pre-diabetes was defined as either impaired fasting glucose or impaired glucose tolerance.

### BP Measurement and Study-Outcome Definitions

Investigators were trained in the measurement of blood pressure and in the questionnaire before the survey according to a standard protocol [19]. The measurement of blood pressure was preformed before OGTT and blood pressure was measured using a standardized mercury sphygmomanometer (regular adult, large, or thigh) in the sitting position after 5 minutes of rest, no smoking, alcohol coffee/tea, exercise and stress at least 30 minutes before examination. Two consecutive readings of blood pressure were taken on the same arm; the mean of the 2 measures was used for analysis. The accuracy rate of blood pressure was 2 millimeter of mercury.

According to Seventh report of the Joint National Committee on Prevention, Detection, Evaluation, and Treatment of High Blood Pressure [20], hypertension was defined as systolic blood pressure (SBP) of 140 mm Hg or greater, diastolic blood pressure (DBP) of 90 mm Hg or greater, or self-reported current treatment for hypertension with antihypertensive medication. Previously diagnosed hypertension was identified by a positive response from the participant to the question, “Has a doctor ever told you that you have hypertension?” Total hypertension included both previously diagnosed hypertension and previously undiagnosed hypertension. The individuals with a normal blood pressure reading at the time of the survey, who had previously been informed by a healthcare professional but were not on treatment, were not considered hypertensive. Awareness of hypertension was defined as self-report of any previous diagnosis of hypertension by a healthcare professional among the population defined as having hypertension. Treatment of hypertension was defined as use of a prescription medication for management of high BP at the time of the interview. Control of hypertension was defined as pharmacological treatment of hypertension associated with an average SBP<140 mm Hg and an average DBP<90 mm Hg. Family history of hypertension was defined by any parental or first-degree sibling history of hypertension.

### Statistical Analysis

All calculations were weighed to represent the total Chinese adult population aged 20 years or older. Weights were calculated based on the Chinese population data from 2006 and the study sampling scheme, which took into account several features of the survey. These features included oversampling for female and urban residents, non-response, economic development, and other demographic or geographic differences between the sample and the total population. Prevalence estimates for hypertension were calculated based on the overall population according to age and sex, in which age- and sex-standardized prevalence was determined using the population distribution of China in 2006 [21]. A multinomial logit analysis was utilized to examine the association between demographic, lifestyle, and metabolic factors with the odds of hypertension. With the use of forward conditional, only covariables that were significant (P<0.05) were retained in the final model. Standard errors were calculated using a technique appropriate for the complex survey design. All P-values were two-tailed with a significance level of 0.05. Statistical analyses were conducted using SUDAAN software (version 10; Research Triangle Institute, Research Triangle Park, NC, USA).

## Results

### Prevalence of Hypertension

**Figure 1:**
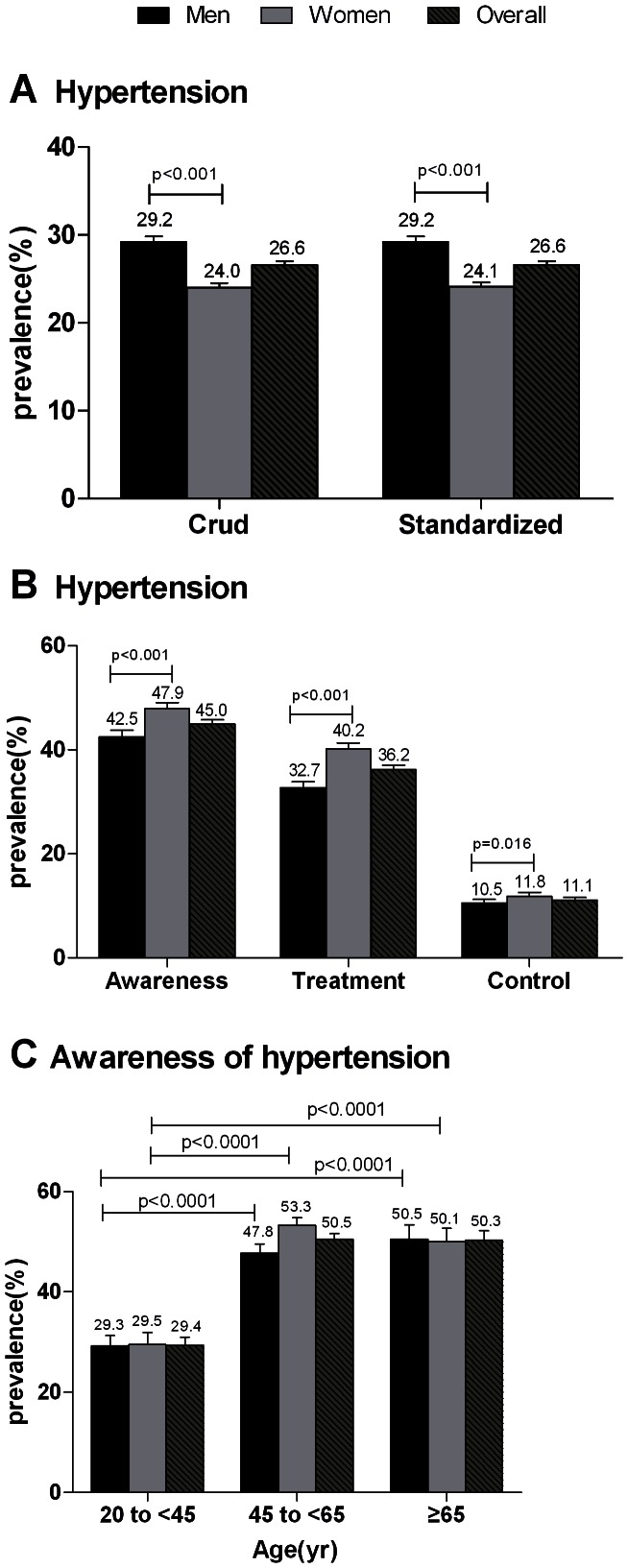
Prevalence, awareness, treatment, and control of hypertension among Chinese aged ≥20 years. (A) The crude and age-standardized prevalence of hypertension by sex. (B) The age-standardized awareness, treatment, and control of hypertension by sex. (C) The awareness of hypertension among different age groups. Bars indicate 95% confidence intervals.

**Figure 2:**
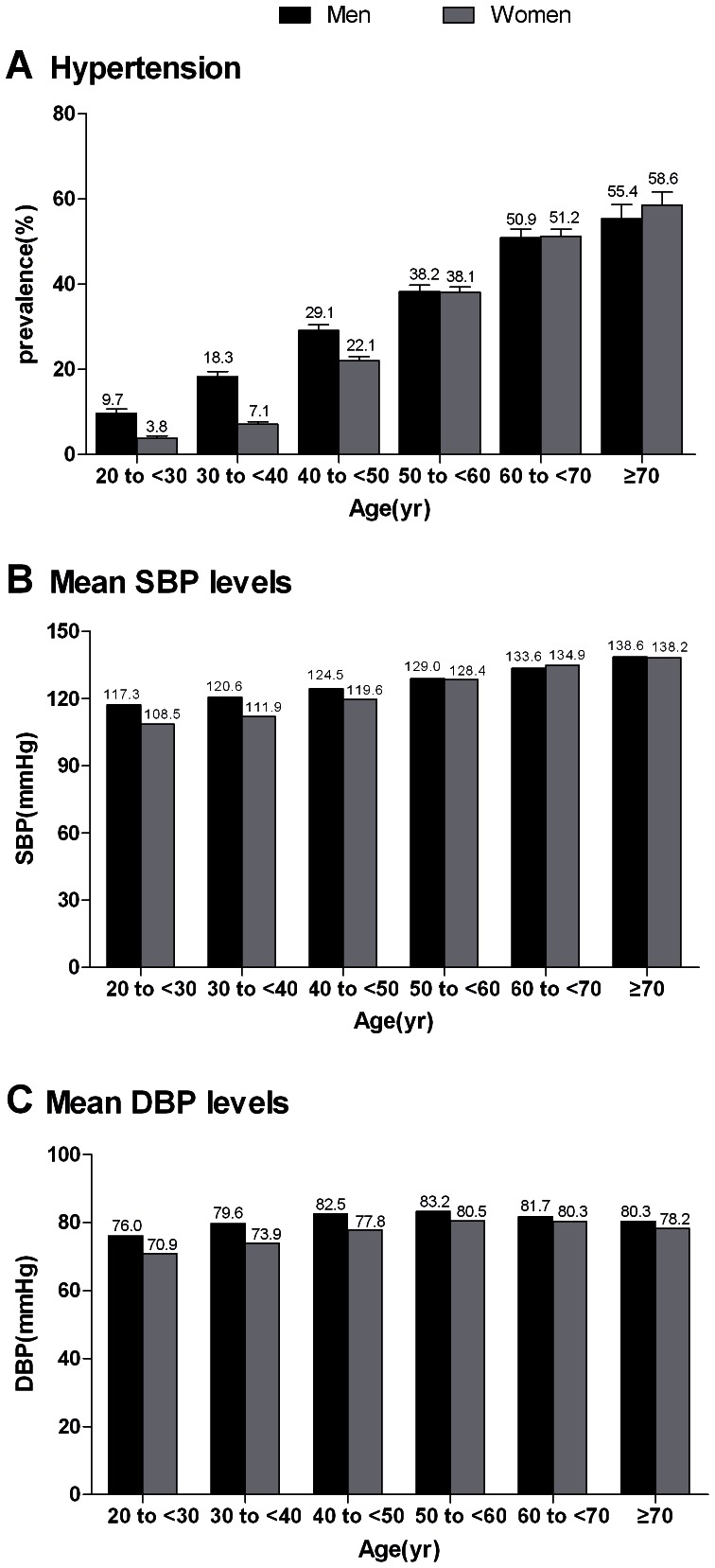
Age-specific prevalence of hypertension and mean blood pressure levels among Chinese aged ≥20 years. The prevalence of hypertension (A), mean systolic blood pressure levels (B) and mean diastolic blood pressure levels (C) among men and women are shown according to age. Bars indicate 95% confidence intervals.

**Figure 3:**
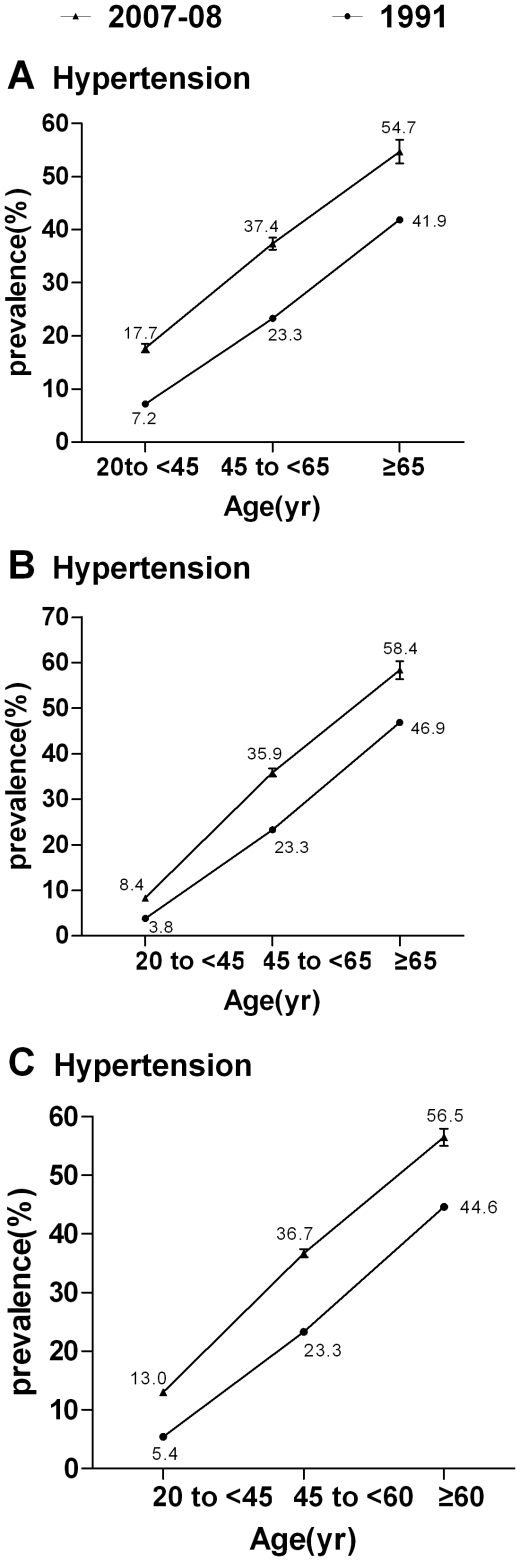
Trends in the prevalence of hypertension among Chinese aged ≥20 years from 1991 to 2007–2008. (A) Trends in the prevalence of hypertension among men from 1991 to 2007–2008 according to different age groups. (B) Trends in the prevalence of hypertension among women. (C) Trends in the prevalence of hypertension in the overall population between 1991 and 2007–2008. Bars indicate 95% confidence intervals.

A total of 26.6% of Chinese adults aged 20 years or older, representing approximately 254 million individuals, had hypertension (Fig. 1A). The age-standardized prevalence of hypertension was significantly higher in men than in women (29.2% vs 24.1%, p<0.001) (Fig. 1A). The age-specific prevalence of hypertension increased with age throughout the entire age range (Fig. 2A). This trend was also observed in mean systolic blood pressure levels by age groups, but not in mean diastolic blood pressure levels (Fig. 2B–C). The age-specific prevalence of hypertension was 13.0%, 36.7%, and 56.5% among persons aged 35 to 44 years (young people), 45 to 64 years (middle-aged people), and ≥65 years (elderly people), respectively (Fig. 3).

### Metabolic Risk Factors

In persons with hypertension, 57.5% of men and 52.1% of women had previously undiagnosed hypertension. Persons with undiagnosed hypertension had significantly lower fasting plasma glucose levels, 2-hour plasma glucose levels, total cholesterol levels, triglyceride levels, low density lipoprotein cholesterol levels, and the insulin resistance index as determined by homeostasis model assessment than did those with diagnosed hypertension. Furthermore, patients with previously undiagnosed hypertension were younger, less likely to have a family history of diabetes and less likely to be obese compared with those with diagnosed hypertension. Therefore, patients with previously undiagnosed hypertension had much less metabolic risk factors than did those with diagnosed hypertension (Table 1).

**Table 1:** Characteristics of Study Participants According to Blood Pressure Categories and Sex*.

	Normotension	Hypertension	Previous undiagnosted hypertension	Previous diagnosted hypertension
Characteristics		Men(N = 18419)		
Participants-no.(%)	12378(70.8%)	6041(29.2%)	3257(16.8%)	2784(12.4%)
Mean systolic blood pressure (95% CI) – mmHg	116.4(116.2–116.6)	143.2(142.8–143.6)	141.9(141.4–142.5)	144.7(144.0–145.4)##
Mean diastolic pressure (95% CI) – mm Hg	75.5(75.4–75.6)	91.4(91.1–91.6)	91.6(91.3–91.9)	91.1(90.6–91.5)##
Mean heart rate (95% CI) – beats/min	72.7(72.5–72.9)	75.3(75.0–75.6)	75.2(74.9–75.6)	75.3(74.9–75.7)
Mean age (95% CI) – yr	41.8(41.6–42.1)	51.4(51.0–51.7)	48.8(48.3–49.2)	54.4(54.0–54.9)##
College or higher level of education (95% CI) – %	28.9(28.1–29.7)	20.8(19.8–21.8)	21.0(19.6–22.4)	20.6(19.1–22.1)
Family history of hypertension (95% CI) – %	28.1(27.3–28.9)	45.6(44.7–47.1)	33.5(31.9–35.1)	59.7(57.9–61.5)##
Mean waist circumference (95% CI) – cm	83.4(83.2–83.5)	89.9(89.6–90.1)	88.5(88.2–88.9)	91.4(91.1–91.8)##
Mean body-mass index (95% CI)	23.7(23.7–23.8)	26.1(26.0–26.2)	25.7(25.5–25.8)	26.6(26.4–26.7)##
Mean total cholesterol (95% CI) – mg/dl	178.4(177.8–179.1)	191.0(190.1–191.9)	190.0(188.8–191.3)	192.1(190.7–193.5)#
Mean triglycerides (95% CI) – mg/dl	142.7(140.8–144.6)	176.8(173.6–180.0)	168.5(164.2–172.7)	186.5(181.6–191.4)##
Mean LDL cholesterol (95% CI) – mg/dl	104.3(103.7–104.9)	114.3(113.4–115.3)	112.6(111.3–113.9)	116.2(114.8–117.6)##
Mean HDL cholesterol (95% CI) – mg/dl	49.0(48.8–49.3)	49.2(48.8–49.5)	49.9(49.5–50.4)	48.3(47.8–48.8)##
Mean fasting plasma glucose (95% CI) – mg/dl	95.0(94.6–95.4)	104.0(103.2–104.7)	102.4(101.4–103.4)	105.8(104.6–106.9)##
Mean 2-hr plasma glucose in oral glucose-tolerance test (95% CI) – mg/dl	116.0(115.0–117.0)	142.8(141.0–144.6)	135.6(133.2–137.9)	151.3(148.5–154.1)##
LogHOMA-IR†	0.48(0.46–0.49)	0.71(0.70–0.73)	0.67(0.65–0.70)	0.76(0.74–0.79)##
LogIAUC‡	3.99(3.98–4.00)	4.17(4.15–4.19)	4.12(4.09–4.15)	4.23(4.20–4.26)##
		**Women(N = 27820)**		
Participants-no.(%)	20665(76.0%)	7155(24.0%)	3319(12.5%)	3836(11.5%)
Mean systolic blood pressure (95% CI) – mmHg	112.3(112.2–112.5)	144.5(144.1–144.9)	143.9(143.4–144.4)	145.1(144.5–145.7)#
Mean diastolic pressure (95% CI) – mm Hg	72.9(72.7–73.0)	88.2(88.0–88.5)	89.2(88.9–89.5)	87.4(87.0–87.8)##
Mean heart rate (95% CI) – beats/min	74.5(74.4–74.7)	76.2(75.9–76.5)	76.5(76.1–76.9)	75.9(75.5–76.2)#
Mean age (95% CI) – yr	41.7(41.5–41.9)	54.4(54.1–54.6)	51.9(51.5–52.3)	56.5(56.2–56.8)#
College or higher level of education (95% CI) – %	23.6(23.0–24.2)	8.5(7.9–9.2)	9.1(8.1–10.1)	8.0(7.1–8.9)##
Family history of hypertension (95% CI) – %	32.2(31.6–32.8)	49.0(47.8–50.2)	35.9(34.3–37.6)	60.3(58.8–61.9)##
Mean waist circumference (95% CI) – cm	77.3(77.2–77.4)	85.4(85.1–85.6)	83.9(83.6–84.2)	86.6(86.3–86.9)##
Mean body-mass index (95% CI)	23.2(23.1–23.2)	25.9(25.8–26.0)	25.4(25.2–25.5)	26.4(26.2–26.5)##
Mean total cholesterol (95% CI) – mg/dl	177.2(176.7–177.7)	198.5(197.6–199.4)	196.1(194.8–197.4)	200.7(199.4–201.9)##
Mean triglycerides (95% CI) – mg/dl	115.9(114.8–117.0)	158.8(156.4–161.2)	149.8(146.3–153.3)	166.6(163.3–169.9)##
Mean LDL cholesterol (95% CI) – mg/dl	102.0(101.6–102.5)	118.4(117.4–119.3)	115.7(114.4–117.1)	120.3(119.1–121.6)##
Mean HDL cholesterol (95% CI) – mg/dl	53.5(53.3–53.7)	53.0(52.7–53.3)	53.0(52.6–53.5)	53.0(52.6–53.4)
Mean fasting plasma glucose (95% CI) – mg/dl	93.2(92.9–93.5)	105.0(104.2–105.7)	102.9(101.8–104.0)	106.7(105.6–107.8)##
Mean 2-hr plasma glucose in oral glucose-tolerance test (95% CI) – mg/dl	115.8(115.1–116.4)	151.9(150.2–153.7)	143.5(141.1–146.0)	159.2(156.7–161.7)##
LogHOMA-IR†	0.47(0.46–0.48)	0.73(0.71–0.74)	0.67(0.65–0.69)	0.78(0.76–0.80)##
LogIAUC‡	4.12(4.11–4.13)	4.26(4.24–4.28)	4.21(4.19–4.24)	4.31(4.28–4.33)##

* Blood pressure level was categorized as follows: normotension (systolic blood pressure, <140 mmHg, diastolic blood pressure, <90 mmHg and without taking antihypertensive medication); hypertension (systolic blood pressure, ≥140 mmHg, diastolic blood pressure, ≥90 mmHg or taking antihypertensive medication). To convert the values for glucose to millimoles per liter, multiply by 0.05551. To convert the values for cholesterol to millimoles per liter, multiply by 0.02586. To convert the values for triglycerides to millimoles per liter, multiply by 0.01129. 95% CI: 95% confidence interval; HDL: high-density lipoprotein; LDL: low-density lipoprotein.

† HOMA-IR: homeostasis of minimal assessment of insulin resistance was calculated from fasting plasma glucose(FPG)and fasting insulin level (FINS)using the following formula; (FPG[mmol/L]×FINS)/22.5.

‡ IAUC: Insulin area under-curve was calculated from FINS, 0.5-hour insulin level (INS0.5 h)and 2-hour glucose level (INS2h)in an oral glucose-tolerance test using the following formula; FINS/4+INSlh+3×INS2h/4.

# p<0.05,

## p<0.001, compared with subjects with Previous undiagnosted hypertension.

### Economic Development, Urbanization, and Geographic Region

**Figure 4:**
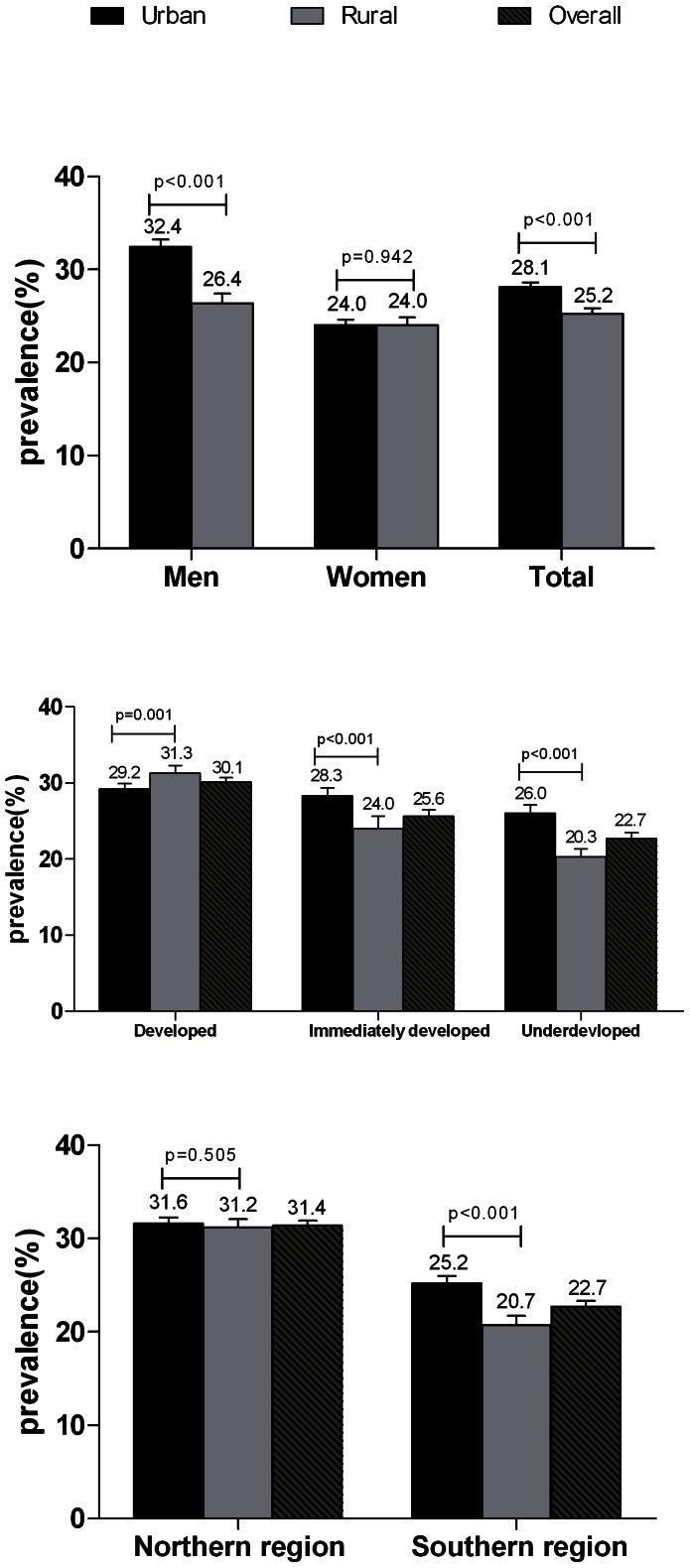
Prevalence of hypertension according to sex, level of economic development, and geographic region among Chinese aged ≥20 years. The prevalence of hypertension among urban and rural residents is shown according to sex (A), level of economic development (B) and geographic region (C). Bars indicate 95% confidence intervals.

Although the prevalence of hypertension was significantly higher in urban residents than in rural residents (28.1% vs. 25.2%, p<0.001), there was no significant difference in the prevalence of hypertension between urban and rural women (24.0% vs. 24.0%, p = 0.942) (Fig. 4A). The increasing prevalence of hypertension was positively correlated with the level of economic development. However, in economically developed regions, the prevalence of hypertension was higher among rural residents than among urban residents (31.3% vs 29.2%, p = 0.001) (Fig. 4B). When 14 provinces, municipalities, and autonomous regions selected in this study were divided into northern and southern China along the boundary of the Qinling Mountains and Huaihe River, residents in the northern region had a higher prevalence of hypertension than those in the southern region (31.4% vs. 22.7%, p<0.001) (Fig. 4C). However, among individuals who lived in the northern region, the prevalence of hypertension in rural residents was comparable with that in urban residents (31.6% vs. 31.2%, p = 0.505) (Fig. 4C).

### Awareness, Treatment, and Control of Hypertension

Overall, 45.0% of those with hypertension were aware of their diagnosis, 36.2% were taking prescribed medication to lower their blood pressure (equivalent to 80.4% of those aware of their condition), and only 11.1% achieved blood pressure control (equivalent to 24.6% of those aware of their condition) (Fig. 1B). More women were aware of their hypertension than men were (47.9% vs. 42.5%, p<0.001). Treatment and control were also more common among women than among men (Fig. 1B). The proportion of awareness of hypertension in affected young people was far lower than middle-aged or elderly people (29.4% vs. 50.5% p<0.001; 29.4 vs. 50.3% p<0.001) (Fig. 1C).

### Multivariable Risk Assessment

In multivariable, binary, logit models, the prevalence of hypertension was positively associated with male sex, older age, a family history of hypertension, educational level below college, increased heart rate, overweight, obesity, central obesity, prediabetes, diabetes, elevated serum triglyceride levels, elevated serum cholesterol levels, a higher level of economic development, and residents living in the northern region, whereas there was an inverse correlation between urban residence and hypertension. Additionally, the log insulin resistance index as determined by homeostasis model assessment and log incremental area under the curve were related to an increased risk of hypertension (Table 2).

**Table 2:** Multivariable-Adjusted Odds Ratios and 95% confidence interval (95% CI) for Hypertension among Men and Women*.

	Hypertension	
Variable	Odds Ratio (95% CI)	P Value
Male sex	1.66(1.56–1.76)	<0.001
Age, per 10-yr increment	1.81(1.76–1.85)	<0.001
Family history of hypertension	2.09(1.97–2.21)	<0.001
Less than college education	1.36(1.26–1.47)	<0.001
Heart rate, per increase of 10 beats/min	1.22(1.19–1.25)	<0.001
Overweight†	1.80(1.1.68–1.93)	<0.001
Obesity‡	3.29(2.94–3.68)	<0.001
Central obesity§	1.41(1.31–1.51)	<0.001
Prediabetes	1.46(1.36–1.57)	<0.001
Diabetes	1.58(1.43–1.73)	<0.001
Triglycerides, per increase of 50 mg/dl (0.56 mmol/liter)	1.04(1.02–1.05)	<0.001
Total cholesterol,per increase of 20 mg/dl (0.52 mmol/liter)	1.08(1.06–1.10)	<0.001
Urban residence	0.92(0.86–0.97)	0.004
Northern region	1.15(1.08–1.23)	<0.001
Immediately developed region	1.36(1.25–1.48)	<0.001
Developed region	1.35(1.26–1.46)	<0.001
LogHOMAIR¶	1.20(1.14–1.27)	<0.001
LogIAUC‖‖	1.12(1.07–1.17)	<0.001

* Odds ratios were calculated with the use of binary logit model (Forward conditional). All covariables listed were included in the model simultaneously.

† Overweight was defined as a body-mass index between 25.0 and 29.9.

‡ Obesity was defined as a body-mass index of 30.0 or more.

§ Central obesity was defined as a waist circumference of 90 cm or more in men and as 80 cm or more in women.

¶ HOMAIR: Homeostasis of minimal assessment of insulin resistance was calculated from fasting plasma glucose (FPG) and fasting insulin level (FINS) using the following formula; (FPG[mmol/L]×FINS)/22.5.

‖‖ IAUC: Insulin area under-curve was calculated from FINS, 0.5-hour insulin level (INS0.5 h) and 2-hour glucose level (INS2 h)in an oral glucose-tolerance test using the following formula; FINS/4+INSlh+3×INS2 h/4.

## Discussion

Findings from the 2007–08 China National Diabetes and Metabolic Disorders Study indicated that 26.6% or 2.54 million of Chinese adults aged ≥20 years had hypertension, of which the prevalence rose by 83.4% since 1991 [5] (14.5% in 1991 vs. 26.6% in 2007–2008). Hypertension is still prevalent worldwide. Data from the National Health and Nutrition Examination Survey (NHANES) indicated that the prevalence rate of hypertension had increased from 24.0% in 1988–1991 [22] to 33.5% in 2005–2008 [23] among US adults. There are likely to be multiple reasons for this increase. An increase in obesity may be the main contributing factor [7], [24]. In our study, obesity was the factor most strongly and independently correlated with hypertension.

Among all hypertensive individuals, nearly half were aware of their condition, more than one third were receiving prescribed antihypertensive medications (equivalent to 80.4% of those aware of their condition), and one ninth had achieved adequate control of blood pressure (equivalent to 24.6% of those aware of their condition). These findings are higher than those reported by the China NNHS 2002 [7], which estimated that the awareness, treatment, and control of hypertension was 24%, 20%, and 5% in individuals, respectively. Moreover, from 2002–2008, the ratio of controlled to treated hypertension also rose from 1∶4.2 to nearly 1∶3. These findings suggest that China has made some progress in the management of hypertension over the past years. However, the awareness, treatment, and control of hypertension from the current study are still substantially lower than those reported by the NHANES 1999–2010 [25], where in hypertensive individuals, 74.0% were aware of their condition, 71.6% were taking antihypertensive medications, 46.5% achieved adequate control of blood pressure, and 64.4% achieved adequate control in treatment. Therefore, China needs to continue promoting community-based standardized blood pressure management programs to further raise the awareness of the condition and the efficacy of treatment. A more aggressive blood pressure-lowering goal and strategy should be established and used.

The trend of an increase in the prevalence of hypertension was much greater in younger age groups compared with older age groups over the past few decades. In men aged <45 years, the prevalence of hypertension in 2007–2008 was almost 2.5 times higher than that in 1991 [5]. Similar increases in prevalence were observed in women aged <45 years. Of more concern, the affected young people had a markedly lower awareness of their condition than did their older counterparts (aged ≥45 years). In the present study, patients with previously undiagnosed hypertension had much less metabolic risk factors compared with those with diagnosed hypertension. This resulted in less chance for early detection and early treatment of hypertension, and partly accounted for the lower awareness among hypertensive patients aged <45 years. Actually, it is well known that young people often live a stressful life, but do not pay enough attention to their health, which may contribute to the rapid growth in the prevalence of hypertension. Therefore, health education and periodic physical examinations may be more effective and important measures to detect hypertension early in such a population.

The gap in the prevalence of hypertension between urban and rural areas was not further narrowed in this survey compared with the data from the China NNHS 2002 [7]. However, in economically developed regions, rural residents had a higher prevalence of hypertension than urban residents. Among women or individuals who lived in the northern region, the disparity in the prevalence of hypertension between urban and rural areas disappeared. Multivariable regression analysis showed that urban residence was negatively correlated with hypertension after multivariable adjustment. We also found similar trends in obesity-related diseases, such as pre-diabetes, which was slightly lower among urban residents than among rural residents (15.0% vs. 16.0%, p = 0.06) [15]. With economic development in rural regions, urbanization and associated lifestyle and diet may explain the convergence in the prevalence of hypertension between persons who live in urban settings and those who live in rural areas [26]. As a country with a vast agricultural base, China should pay more attention to improving the health status of rural residents.

The current study has potential limitations, some of which have been mentioned in previous studies by our group [15]. First, dietary intake and work-related physical activity were not assessed in our study. Therefore, we were not able to determine the association between these factors and the prevalence of hypertension. Second, because we are unable to obtain the original data of the population samples in 1991 and 2002, mutual comparison of the characters of the three population samples (1991, 2002 and 2007–2008) could not be carried out. However, the sampling methods among these population samples were similar, and the prevalence of hypertension from the three population samples reflects the national epidemic status in their respective time periods. Therefore, the changing trends from 1991–2008 can still be shown through the mutual comparison of the prevalence of hypertension among the three population samples.

In conclusion, the present study indicates that the prevalence of hypertension in China is increasing. Although hypertension awareness, treatment, and control have improved during the past several years, the overall rates remain poor. Public health efforts for further improving awareness and enhancing effective control are still urgently required in China. In addition, the trend of an increase in the prevalence of hypertension is much greater in young people and rural populations, notwithstanding elevated blood pressure being pervasive in various age groups and across all socioeconomic groups. These emerging populations should be focused on in community-based standardized blood pressure management programs in China.
